# RNA virus evasion of nonsense-mediated decay

**DOI:** 10.1371/journal.ppat.1007459

**Published:** 2018-11-19

**Authors:** Jared P. May, Xuefeng Yuan, Erika Sawicki, Anne E. Simon

**Affiliations:** 1 Department of Cell Biology and Molecular Genetics, University of Maryland–College Park, College Park, Maryland, United States of America; 2 College of Plant Protection, Shandong Agricultural University, Taian, Shandong Province, P.R.China; Agriculture and Agri-Food Canada, CANADA

## Abstract

Nonsense-mediated decay (NMD) is a host RNA control pathway that removes aberrant transcripts with long 3’ untranslated regions (UTRs) due to premature termination codons (PTCs) that arise through mutation or defective splicing. To maximize coding potential, RNA viruses often contain internally located stop codons that should also be prime targets for NMD. Using an agroinfiltration-based NMD assay in *Nicotiana benthamiana*, we identified two segments conferring NMD-resistance in the carmovirus *Turnip crinkle virus* (TCV) genome. The ribosome readthrough structure just downstream of the TCV p28 termination codon stabilized an NMD-sensitive reporter as did a frameshifting element from umbravirus *Pea enation mosaic virus*. In addition, a 51-nt unstructured region (USR) at the beginning of the TCV 3’ UTR increased NMD-resistance 3-fold when inserted into an unrelated NMD-sensitive 3’ UTR. Several additional carmovirus 3’ UTRs also conferred varying levels of NMD resistance depending on the construct despite no sequence similarity in the analogous region. Instead, these regions displayed a marked lack of RNA structure immediately following the NMD-targeted stop codon. NMD-resistance was only slightly reduced by conversion of 19 pyrimidines in the USR to purines, but resistance was abolished when a 2-nt mutation was introduced downstream of the USR that substantially increased the secondary structure in the USR through formation of a stable hairpin. The same 2-nt mutation also enhanced the NMD susceptibility of a subgenomic RNA expressed independently of the genomic RNA. The conserved lack of RNA structure among most carmoviruses at the 5’ end of their 3’ UTR could serve to enhance subgenomic RNA stability, which would increase expression of the encoded capsid protein that also functions as the RNA silencing suppressor. These results demonstrate that the TCV genome has features that are inherently NMD-resistant and these strategies could be widespread among RNA viruses and NMD-resistant host mRNAs with long 3’ UTRs.

## Author summary

Nonsense-mediated decay (NMD) serves a critical role in preserving the integrity of the host transcriptome. RNA transcripts bearing premature termination codons (PTCs) are subject to NMD as they can lead to expression of truncated, deleterious proteins that are associated with many genetic diseases and cancer. Long 3’ UTRs are known to promote NMD, but many transcripts that naturally contain long 3’ UTRs are protected. The nature and possible commonality of this protection is not known aside from sequences just downstream from termination codons that bind polypyrimidine tract binding protein 1. Natural stop codons in the genomes of multicistronic RNA viruses would resemble PTCs and thus these viruses must have developed the means to combat NMD to maintain genome stability. We show that the compact, positive-sense *Turnip crinkle virus* evades NMD by several strategies including having an unstructured region of RNA immediately downstream of the coat protein (CP) termination codon. CP is a critical protein in the virus lifecycle since CP is required to both inhibit host antiviral RNA silencing and form virions. We provide evidence that this 51-nt unstructured sequence can protect NMD-sensitive templates from NMD when placed just downstream of stop codons. Unstructured regions following stop codons were over 4-fold enriched in a set of >6000 human NMD-resistant transcripts versus *bona fide* UPF1 targets (NMD-sensitive), suggesting this may be a wide-spread strategy for protecting otherwise susceptible transcripts from NMD.

## Introduction

Since viruses are obligate intracellular parasites, host cellular RNA control pathways must be tolerated or circumvented for successful virus amplification. One such pathway is nonsense-mediated decay (NMD), which normally removes aberrant mRNAs containing premature termination codons (PTCs) to prevent detrimental effects caused by the expression of truncated proteins. The mechanism of NMD has been extensively studied for PTC-containing mRNAs with exon-junction complexes (EJCs) deposited during splicing downstream of the PTC (see [1] for a review). The EJC is associated with NMD factors and is removed by the ribosome-associated PYM protein during normal translation, but remains unaltered in PTC-containing mRNAs, which leads to NMD [2]. Key NMD factors that are conserved across nearly all eukaryotes [3] include the Up-frameshift group of proteins (UPF1-3), with UPF1 as the master regulator [4]. UPF1 contains ATPase and RNA helicase domains necessary for NMD and is regulated by a complex involving UPF2 and UPF3 [5, 6]. Activated UPF1 recruits nucleases to eliminate NMD-targeted transcripts, with key differences existing between mammalian and plant systems [7]. For example, both the SMG6 endo- and SMG7-guided exonucleolytic pathways are utilized in mammalian cells [8, 9], whereas only SMG7-coordinated exonucleolytic cleavage is used in plants due to the absence of SMG6 homologs [10, 11].

In addition to EJC-dependent NMD caused by 3’ UTR introns and upstream open reading frames (uORFs), EJC-independent NMD has been described for mRNAs containing long 3’ UTRs or uORFs [12]. For polyadenylated mRNAs with short 3’ UTRs, translation termination includes an interaction between eukaryotic release factor 3 (eRF3) and cytoplasmic poly-A binding protein (PABPC1), which restricts UPF1 binding. Long 3’ UTRs limit eRF3-PABPC1 interactions, allowing elevated levels of UPF1 to associate with the termination complex and increasing the likelihood of UPF1 initiating the NMD pathway [13–18]. Multiple genome-wide searches, however, have identified a large number of genes with exceptionally long 3’ UTRs that are resistant to NMD [19–22], but few have defined mechanisms for NMD-evasion. Human polypyrimidine tract binding protein 1 (PTBP1) has been identified as an NMD-antagonist by binding polypyrimidine hexamers in the vicinity of NMD-targeted termination codons, which prevents NMD by displacing UPF1 [23]. In yeast, the RNA binding protein Pub1 protects the GCN4 and YAP1 mRNAs from NMD by direct binding to AU-rich elements downstream of their respective uORFs [24].

Most RNA viruses have multicistronic genomes to conserve limited encapsidation space. As a result, many viral genomic (g)RNAs, especially those associated with 3’-co-terminal subgenomic (sg)RNAs, are templates for translation of only the 5’ proximal ORF leaving a long 3’ UTR (e.g., >3000 nt). Since this translation strategy makes these viruses optimal targets for NMD, most have likely evolved strategies to evade degradation [25, 26]. However, only three such strategies have been elucidated. For example, similar to some resistant mRNAs, *Rous sarcoma virus* (RSV) contains a polypyrimidine-rich *cis*-acting sequence immediately downstream of the *gag* termination codon that provides protection of an otherwise optimal target (>6700-nt 3’ UTR) by recruiting PTBP1 [23, 27, 28]. In addition, the readthrough elements from *Moloney murine leukemia virus* and *Colorado tick fever virus* inhibit NMD by allowing ribosomes to continue translation past the termination codon [29, 30]. Finally, global-inhibition of NMD has been described for *Human T-lymphotropic Virus Type 1* (HTLV-1) and *Hepatitis C virus* (HCV). The HTLV-1 Tax and Rex proteins inhibit NMD through binding to UPF1 [31, 32] while the HCV core protein inhibits NMD by disrupting interactions between NMD-associated proteins WIBG, Y14, and Magoh [33]. Elucidating additional NMD-evasion strategies employed by viruses, especially strategies that could be more widespread, is not only important for understanding virus fitness but also for providing insights into additional mechanisms used by NMD-resistant cellular mRNAs.

**Fig 1:**
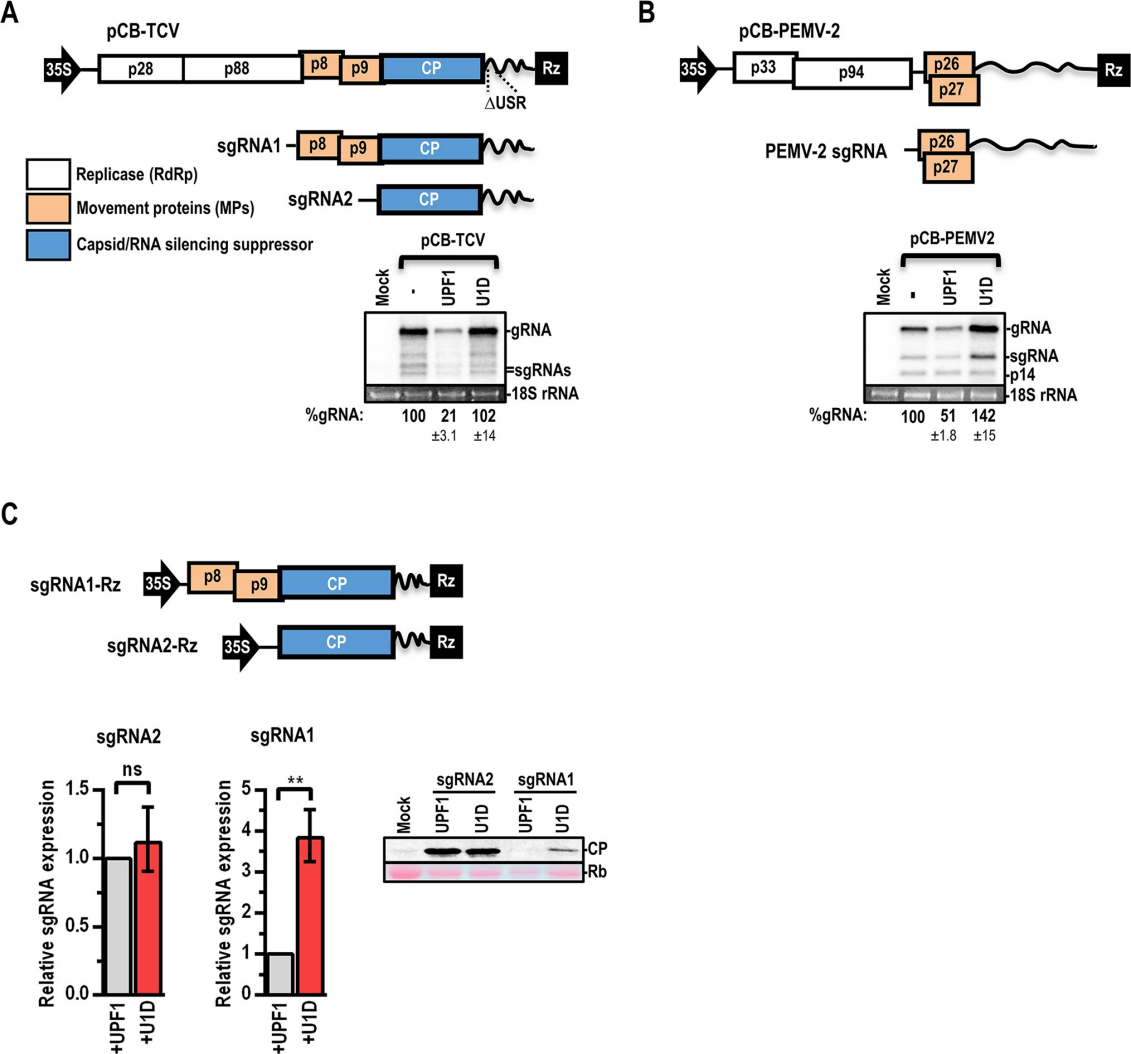
TCV and PEMV-2 display varying levels of NMD sensitivity. (**A**) Genomic organization of TCV. p28 and p88 are translated from the gRNA. p8 and p9 are translated from sgRNA1 and CP is predominantly translated from sgRNA2. pCB-TCV was agroinfiltrated into *N*. *benthamiana* alone (-), or together with UPF1 or U1D (dominant-negative UPF1 that inhibits NMD). At 72 hpi, leaf infiltration spots were collected for RNA extraction and northern blotting. gRNA levels were normalized to those of 18S rRNA and TCV-WT levels were set to 100%. Data are from three independent leaves and standard deviations are shown. (**B**) Genomic organization of PEMV-2. p33 and p94 replicase proteins are translated from the gRNA while p26 and p27 movement-associated proteins are translated from the sgRNA. PEMV-2 was assayed for NMD sensitivity in the same manner as TCV but was also co-expressed with the p14 silencing suppressor since PEMV-2 does not encode a suppressor protein. (**C**) TCV sgRNA1 and sgRNA2 were co-expressed with either UPF1 or U1D. mRNA accumulation was assayed by RT-qPCR and CP levels were determined by western blotting. RT-qPCR data are from at least 5 independent leaves and levels were normalized to those of p14. Protein levels are representative of two independent leaves. Rb, Ribulose bis-phosphate carboxylase. Paired t test: ** *p*<0.01.

*Turnip crinkle virus* (TCV) has a positive-sense, single-stranded gRNA (4053 nt) and two sgRNAs that together code for 5 proteins (Fig 1A). When translation of the gRNA terminates at the end of the 5’ p28 ORF, the 3’ UTR is >3200 nt in length. Infrequently, ribosomes read through the p28 stop codon to translate the p88 RNA-dependent RNA polymerase (RdRp). p8 and p9 are translated from sgRNA1 by leaky scanning [34] and are important for virus cell-to-cell movement. The p38 CP, which is predominantly expressed from sgRNA2, requires synthesis throughout the infection cycle as CP serves as both the virus’s RNA silencing suppressor [35] and as the sole protein required for capsid formation (180 copies/virion). The 3’ UTR of sgRNA2 (254 nt) is considered short compared to some viruses in the *Tombusviridae*, but average compared to 3’ UTRs of host mRNAs (average 3’ UTR length in *Arabidopsis* is 236 nt; see S1 Fig). The 3’ UTRs of viruses in the *Tombusviridae* have significantly higher GC% content compared to host mRNAs (S1 Fig), and elevated GC% content has been associated with increased UPF1 association and NMD [36]. Together, the multicistronic organization and high-GC% content of the TCV 3’ UTR should predispose TCV gRNA and sgRNAs to NMD. In support of this, an earlier study demonstrated that TCV accumulation was strongly reduced by overexpression of UPF1 compared with levels when NMD was suppressed by addition of U1D, a dominant negative version of UPF1 that is a potent inhibitor of NMD [37, 12]. The authors thus concluded that NMD is a general virus restriction pathway in plants [37]. However, TCV replicates to ribosomal RNA levels in both cell culture and *in planta*, suggesting that TCV must be evading NMD and thus the endogenous (i.e., UPF1-unsupplemented) NMD pathway is probably insufficient to substantially limit TCV accumulation.

In this study, we identified two regions in the TCV genome that antagonize NMD including a 51-nt pyrimidine-rich unstructured region just downstream of the CP ORF. Resistance was maintained when 19 pyrimidines were converted to purines but abolished when a downstream 2-base mutation generated a stable hairpin in the region. An RNA structure that mediates low level ribosome readthrough just past the 5’ proximal ORF also protected NMD-sensitive transcripts. Predicted unstructured RNA regions downstream of termination codons were over 4-fold enriched in >6000 human NMD-resistant transcripts compared with a set of validated UPF1 targets, suggesting that this NMD prevention strategy may be common across host transcriptomes.

## Results

### Viruses in the *Tombusviridae* display varying levels of NMD sensitivity

Two viruses from the *Tombusviridae*, carmovirus TCV and umbravirus *Pea enation mosaic virus 2* (PEMV-2) were assessed for NMD sensitivity. PEMV-2 has an unusually long 3’ UTR (704 nt) and generates a sgRNA with overlapping ORFs that encode proteins p27 and p26 (Fig 1B). p27 and p26 are required for short and long-distance movement of the virus, respectively, and p26 is additionally required for virus accumulation in protoplasts [38]. TCV and PEMV-2 gRNA expression constructs with 3’ end ribozymes (Rz) were introduced into *Nicotiana benthamiana* leaves by agroinfiltration either alone (-), or co-expressed with either UPF1 or the NMD-inhibitor U1D, and RNA levels were determined by northern blotting 72 hours post-infiltration (hpi). As previously reported using the same assay [37], TCV was sensitive to UPF1 overexpression and levels were strongly enhanced when NMD was inhibited by the presence of U1D (Fig 1A). However, when compared with UPF1-unsupplemented levels (-), TCV accumulation was not enhanced by U1D, suggesting that TCV evades NMD during infection. PEMV-2 gRNA levels were also markedly increased by co-expression with U1D compared with overexpression of UPF1 (Fig 1B). Compared with UPF1-unsupplemented levels, PEMV-2 gRNA levels increased by 42% in the presence of U1D and sgRNA levels were substantially higher. These results suggest that PEMV-2 is more sensitive to NMD compared with TCV.

To determine if TCV sgRNAs are resistant to NMD when expressed independent of virus infection, expression constructs containing sgRNA1 or sgRNA2 were co-expressed with either UPF1 or U1D. RT-qPCR was used to detect sgRNA accumulation and CP protein levels were detected by western blotting (CP is expressed at low-levels from sgRNA1 by leaky scanning [34] and upstream IRES activity [39]). sgRNA2 RNA levels and CP levels did not increase when NMD was inhibited by U1D (Fig 1C), indicating that sgRNA2 is either not susceptible to NMD or is able to evade NMD. In contrast, sgRNA1 was sensitive to NMD under these conditions, with levels increasing by nearly 4-fold in the presence of U1D compared to UPF1 (Fig 1C). Surprisingly, sgRNA1 levels were not detectably different during TCV infection when U1D was overexpressed (Fig 1A). A possible explanation is that sgRNA1 produced by virus replication in the cytosol and lacking a 5’ cap is NMD-resistant, whereas sgRNA1 produced via agroinfiltration in the nucleus bearing a 5’ cap is NMD-sensitive.

### TCV 3’ UTR and ribosome recoding region protect GFP reporter transcripts with long 3’ UTRs against NMD

**Fig 2:**
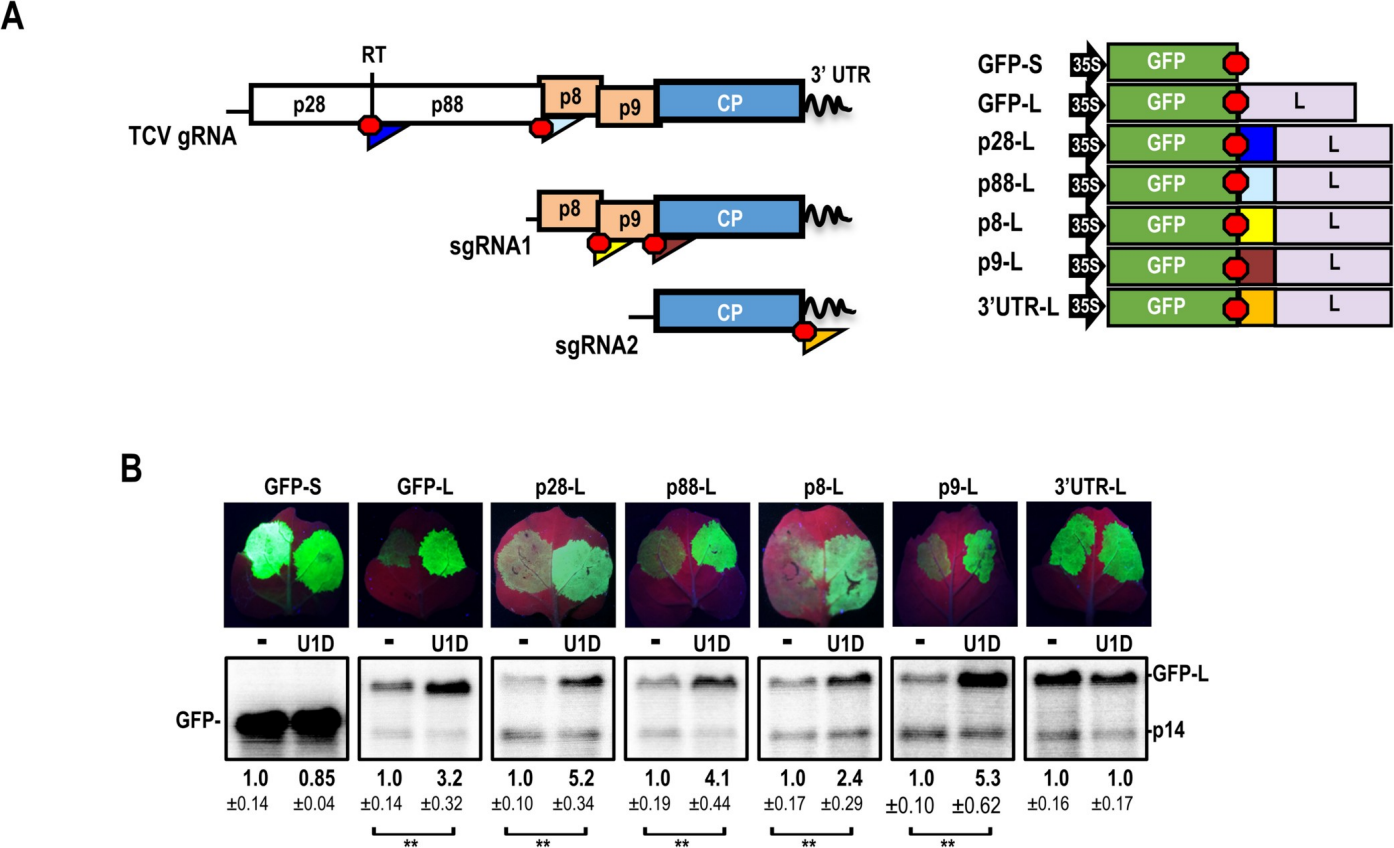
The 3’ UTR of TCV can protect a reporter construct against NMD. (**A**) GFP-reporter constructs used to examine whether the 250 nt downstream of p28, p88, p8, p9, and CP ORFs (color-coded triangles) confer NMD resistance. Red hexagons denote stop codons. TCV segments were inserted just downstream of the GFP ORF in construct GFP-L, which contains the CaMV 35S promoter and a 577-nt “L” segment derived from the bean phytohemagglutinin gene followed by the CaMV 35S terminator sequence. (**B**) GFP reporter constructs were agroinfiltrated with a construct expressing p14 and either without (-) or with a construct expressing U1D. Leaves naturally fluoresce red and infiltrated tissue expressing GFP fluoresce green. RNA was extracted from individual infiltrated “spots” and subjected to northern blot analysis using GFP- and p14-specific oligonucleotide probes. Values were normalized to p14 levels. Data are from three independent infiltrations. Standard deviation is shown. Paired two-tailed t test: ** *p*<0.01.

Genomic organization of TCV necessitates internal stop codons that should be NMD targets since translation termination leaves long 3’ UTRs (Fig 2A). Since a *cis*-acting RNA element just downstream of an internal stop codon in RSV confers resistance to NMD [28], we evaluated whether sequences immediately downstream of each TCV stop codon could provide similar protection. Agroinfiltration was used to deliver transiently expressing green fluorescent protein (GFP) reporter constructs in the absence or presence of co-infiltrated U1D [12]. If added sequences cause normally susceptible transcripts to be protected from NMD, then GFP levels should not differ significantly in the presence or absence of U1D. If transcripts remain sensitive to NMD, then suppression of NMD by U1D would allow those transcripts to accumulate to higher levels. To prevent host RNA silencing from affecting results and to provide for an internal control, the p14 RNA silencing suppressor from *Pothos latent virus* was co-infiltrated in all experiments [40].

As previously reported [12], a GFP reporter construct (GFP-S) containing the GFP ORF followed by the 35S terminator (roughly 182 nt) was resistant to NMD since co-infiltration of U1D did not enhance transcript levels (Fig 2B). In contrast, a GFP reporter construct with a long, 747 nt 3’ UTR derived from the coding region of the bean phytohemagglutinin gene (GFP-L) was NMD-sensitive as addition of U1D increased transcript levels by 3.2-fold (Fig 2B). When 250-nt regions following the stop codons of p28, p88, p8, p9, and CP ORFs were placed just downstream of the GFP stop codon in GFP-L (generating p28-L, p88-L, p8-L, p9-L, and 3’UTR-L, respectively; Fig 2A), only 3’UTR-L transcripts were protected against NMD (Fig 2B). In contrast, GFP mRNA levels for the remaining constructs increased 2.4- to 5.3-fold when NMD was inhibited by U1D, similar to the increase for GFP-L (3.2-fold). This suggests that the sequence just downstream of the CP ORF protects one or more TCV transcripts from NMD during infection.

Translation of the gRNA mainly terminates at the p28 ORF stop codon unless ribosomes read through the stop codon to produce the p88 RdRp [41]. This infrequent recoding event ensures that only a limited amount of RdRp is synthesized, which is important for viral fitness [42, 43]. Since earlier reports revealed that ribosomal readthrough of NMD-sensitive stop codons protects transcripts from NMD (likely through displacement of UPF1 by the translocating ribosome [29, 30, 44]), we noted that construct p28-L, which contains the TCV recoding structural element (RSE) that mediates readthrough [45], did not provide NMD-resistance. The TCV RSE is an extended pseudoknotted hairpin that connects with the Pr hairpin at the gRNA’s 3’ terminus via a long-distance RNA-RNA interaction. The TCV RSE and similar structures found in the vast majority of viruses in the *Tombusviridae* are located just downstream of the 5’ proximal ORF UAG termination codon, incorporating the guanylate as one member of the terminal base-pair of the structure [41]. In contrast, the RSE in p28-L was located 7-nt downstream from the GFP termination codon, which may have prevented sufficient ribosome readthrough. In addition, there was no long-distance interaction with a 3’ proximal hairpin that is critical for efficient readthrough [45]. Furthermore, termination codons in the out-of-frame 3’ UTR “L” sequence downstream of the RSE-containing segment would have caused early termination, likely preventing disassociation of UPF1 by translocating ribosomes.

**Fig 3:**
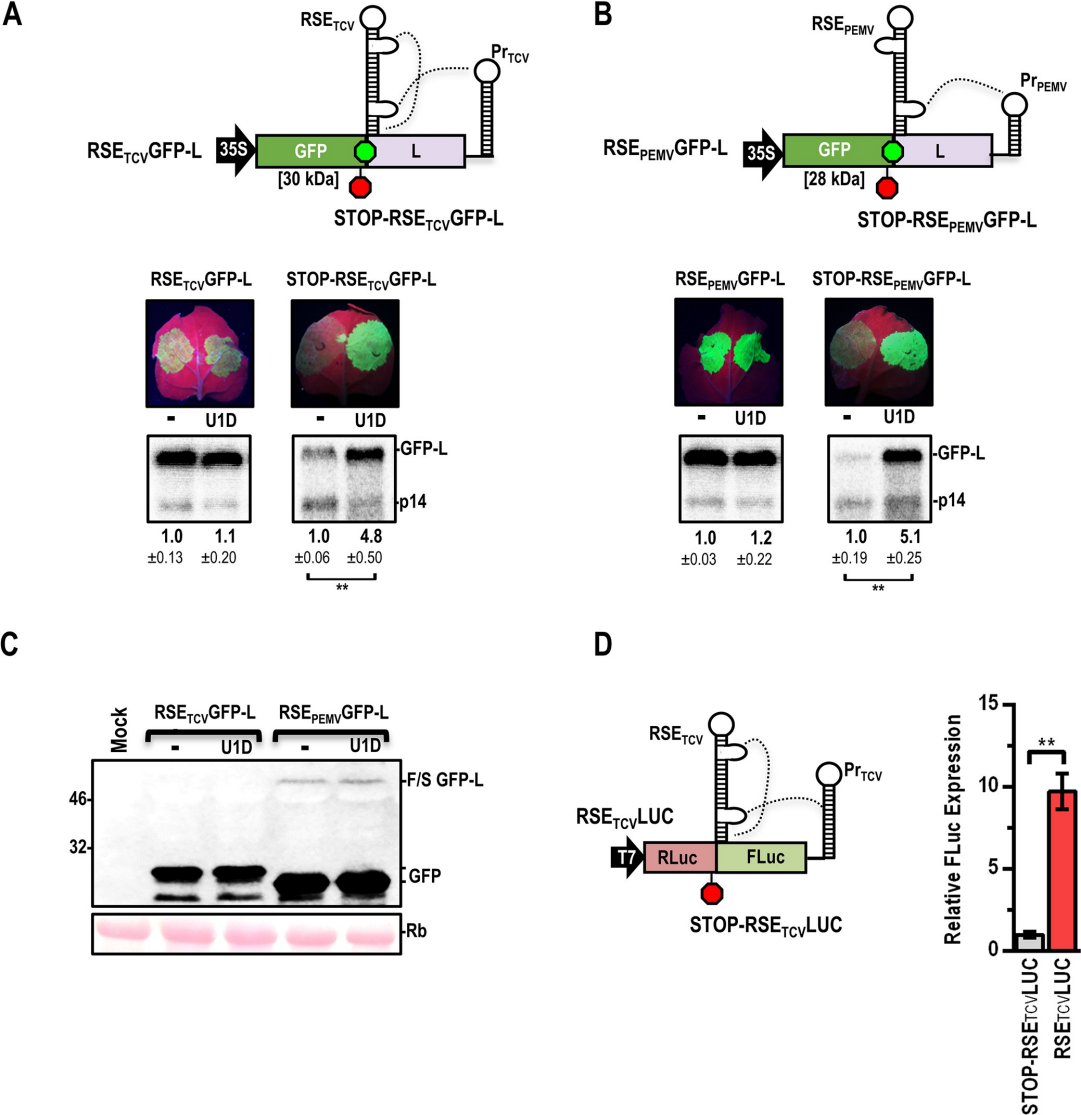
Viral ribosome recoding elements can stabilize NMD-sensitive transcripts. (**A**) Top, TCV ribosome readthrough element (double bulged hairpin), the adjacent stop codon (green hexagon) and an upstream conserved hairpin (not shown) were placed just downstream of the GFP ORF in GFP-L (RSE_TCV_GFP-L) along with the TCV 3’ proximal Pr hairpin. STOP-RSE_TCV_GFP-L contains an additional stop codon (red hexagon) upstream of the RSE. Bottom, accumulation of transcripts in agroinfiltrated *N*. *benthamiana* in the presence and absence of U1D along with p14. (**B**) Top, PEMV-2 ribosome frameshifting element and upstream conserved hairpin (not shown) were placed just downstream of the GFP ORF generating RSE_PEMV_GFP-L. STOP-RSE_PEMV_GFP-L contains an additional stop codon immediately upstream of the RSE cassette. Bottom, results from agroinfiltration. Data from panels A-B are from three independent infiltrations and standard deviation is shown. See legend to Fig 2B for additional information. (**C**) Detection of ribosome-recoded proteins in infiltrated tissue by western blots using a GFP antibody. GFPs expressed from the TCV and PEMV constructs migrate differently as the sequences upstream of their respective RSEs differ in size. Rb, Ribulose bis-phosphate carboxylase. (**D**) Left, RSE_TCV_ and STOP-RSE_TCV_ cassettes were ligated into a dual-luciferase vector containing the TCV 3’ UTR. Right, dual-luciferase RNAs were translated in WGE and relative FLuc expression was determined. STOP-RSE_TCV_LUC FLuc expression was set to 1.0. Data are from four independent samples and standard deviation is shown. Unpaired t test: ** *p*<0.01.

To determine if the TCV RSE protects GFP-L if properly situated, a cassette containing the p28 stop codon, the adjacent RSE, and a conserved hairpin upstream of the RSE was inserted into the NMD-sensitive GFP-L reporter construct replacing the GFP stop codon, generating RSE_TCV_GFP-L (Fig 3A, top). The TCV Pr hairpin was also added at the 3’ end of the construct to provide for the long-distance interaction. As a control, an extra stop codon was inserted just upstream of the cassette within RSE_TCV_GFP-L (construct STOP-RSE_TCV_GFP-L).

Despite proper placement of the RSE, attempts to detect a readthrough product in leaves infiltrated with RSE_TCV_GFP-L were unsuccessful (Fig 3C). Since readthrough efficiencies as low as 1.5% provide substantial resistance to NMD [18, 30], a highly sensitive dual-luciferase reporter was generated with the RSE cassettes inserted between the luciferase ORFs along with the 3’ UTR of TCV to provide for the long-distance interaction (constructs RSE_TCV_LUC and STOP-RSE_TCV_LUC; Fig 3D, left). When translated in wheat germ extracts (WGE), RSE_TCV_LUC generated 10-fold higher expression of firefly luciferase compared with STOP-RSE_TCV_LUC, suggesting that low-level readthrough was occurring when the RSE was properly situated (Fig 3D, right). When RSE_TCV_GFP-L and STOP-RSE_TCV_GFP-L were infiltrated into *N*. *benthamiana*, RSE_TCV_GFP-L transcripts, but not STOP-RSE_TCV_GFP-L transcripts, were nearly completely protected against NMD (Fig 3A, bottom). These results suggest that the TCV RSE provides protection when correctly situated and promoting low-level ribosome recoding.

To further support the importance of low-level ribosome recoding in conferring protection against NMD, we replaced the GFP stop codon in GFP-L with the PEMV-2 RSE (and its upstream hairpin), generating RSE_PEMV_GFP-L. The RSE of PEMV-2 promotes recoding through -1 ribosomal frameshifting, which also requires a long-distance RNA-RNA interaction with a 3’ terminal hairpin that was included in RSE_PEMV_GFP-L [46]. Unlike the TCV RSE, RSE_PEMV_GFP-L transcripts generated sufficient frameshift product in agroinfiltrated leaves to be detected by western blotting (Fig 3C). As with the TCV RSE, RSE_PEMV_GFP-L transcripts, but not STOP-RSE_PEMV_GFP-L transcripts, were protected against NMD (Fig 3B, bottom). Although inefficient at ribosome recoding, we conclude that TCV and PEMV-2 recoding elements are capable of protecting an NMD-sensitive transcript and this mechanism could contribute to protecting their respective gRNAs from NMD in natural infections.

### A polypyrimidine region at the 5’ end of the TCV 3’ UTR is critical for NMD-resistance

**Fig 4:**
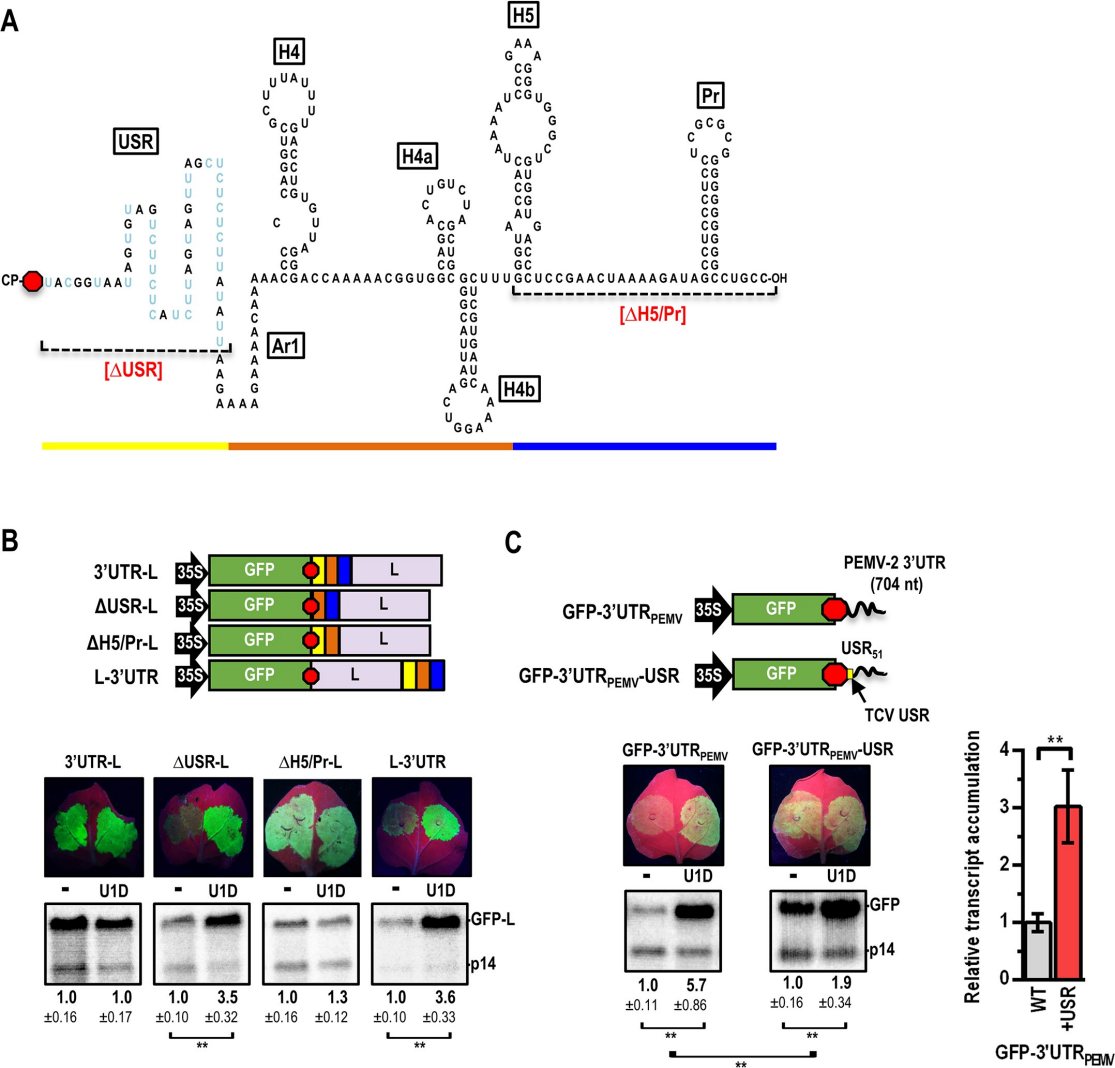
The USR at the 5’ end of the TCV 3’ UTR is critical for NMD-resistance and can protect an unrelated NMD-sensitive transcript. (**A**) Secondary structure of the TCV 3’ UTR. USR pyrimidine residues are colored blue. Deletions that were introduced in the 3’UTR-L reporter are indicated. (**B**) Top, deletion-containing constructs. The 3’ UTR was also relocated to the 3’ terminus of GFP-L (L-3’UTR). Bottom, accumulation of transcripts in agroinfiltrated *N*. *benthamiana* in the presence and absence of U1D along with p14. Data are from three independent infiltrations and standard deviation is shown. See legend to Fig 2B for additional information. (**C**) Top, constructs used to assess if PEMV-2 contains an NMD-protective region in its 3’ UTR. GFP-3’UTR_PEMV_, the 3’ UTR of PEMV-2 was placed downstream of GFP. GFP-3’UTR_PEMV_-USR, the first 51 nt of the PEMV-2 3’ UTR were replaced by the analogous segment of the TCV 3’ UTR (yellow). Bottom, accumulation of transcripts in agroinfiltrated *N*. *benthamiana* in the presence and absence of U1D along with p14. Data are from six leaves and standard deviation is shown. See legend to Fig 2B for additional information. Fold-increase in RNA accumulation with the USR was determined by comparing the fold-increase with U1D between GFP-3’UTR_PEMV_ and GFP-3’UTR_PEMV_-USR.

We previously determined that the TCV 3’ UTR contains an unstructured region (USR) at its 5’ end that is important for some aspect of virus accumulation [47], and a 3’ region with structures required for replication and translation [48] (Fig 4A). To determine which portion of the TCV 3’ UTR was responsible for protecting GFP-L against NMD in construct 3’UTR-L, deletions were constructed within the 3’ UTR sequence in 3’UTR-L (Fig 4B, top). Deletion of the 3’ terminal 91 nt (ΔH5/Pr-L) did not affect NMD-resistance, whereas deletion of the 51-nt USR (ΔUSR-L) abolished protection against NMD (Fig 4B, bottom). When the complete 3’ UTR was positioned 578 nt downstream of the GFP stop codon (and just upstream of the 35S terminator sequence) in construct GFP-L (generating L-3’UTR), transcripts were no longer protected against NMD. These results suggest that the region at the beginning of the TCV 3’ UTR provides NMD-resistance when located just downstream of a stop codon.

When the PEMV-2 3’ UTR was placed downstream of the GFP ORF (construct GFP-3’UTR_PEMV_), transcripts accumulated 5.7-fold higher in the presence of U1D (Fig 4C), suggesting that PEMV-2 does not contain a comparable protective region at the 5’ end of its 3’ UTR. To determine if the TCV USR alone can provide NMD-resistance, the 51-nt TCV USR was substituted for the first 51-nt of the PEMV-2 3’ UTR in the reporter construct generating GFP-3’UTR_PEMV_-USR (Fig 4C). The presence of the 51-nt USR stabilized transcripts by 3-fold, but did not confer complete protection as transcript levels in the presence of U1D were still 1.9-fold higher than in the absence of U1D (Fig 4C). These results indicate that the TCV 51-nt USR alone is capable of providing partial NMD-resistance when placed immediately downstream of an NMD-targeted stop codon.

### The 3’ UTR of a related carmovirus confers protection against NMD

**Fig 5:**
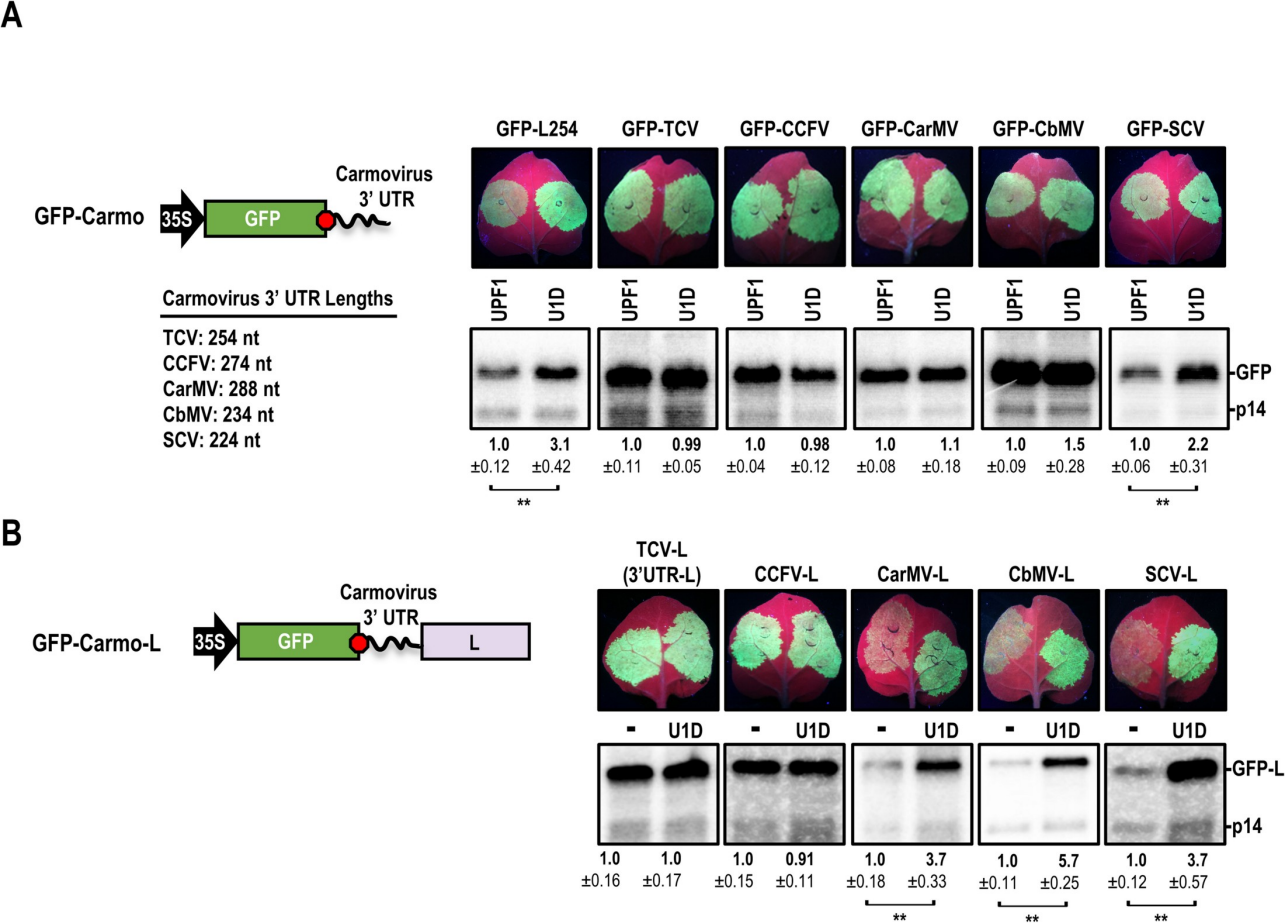
3’ UTRs of TCV and CCFV confer efficient protection against NMD. (**A**) TCV, CCFV, CarMV, CbMV, and SCV 3’ UTRs were placed downstream of GFP. A 254-nt sequence derived from the “L” ORF was used as a control (GFP-L254). Constructs were agroinfiltrated with constructs expressing either UPF1 or U1D along with p14. Only GFP-L254 and GFP-SCV showed increased RNA accumulation with U1D. (**B**) The carmovirus 3’ UTRs were inserted into GFP-L to determine if strong NMD-resistance could be conferred. Constructs were agroinfiltrated with constructs expressing either mock (-) or U1D along with p14. Data from both panels are from three infiltrated leaves. Standard deviation is shown. See legend to Fig 2B for more information.

The 254 nt TCV 3’ UTR was placed downstream of GFP ORF in GFP-S to determine if NMD-resistance is conferred in a context where the TCV 3’ UTR is the only UTR sequence upstream of the 35S terminator (construct GFP-TCV; Fig 5A, top). As a control, GFP-L254 was constructed containing a random 254-nt segment derived from the L sequence. UPF1 was over-expressed to increase the potency of NMD and provide for a more stringent assessment of NMD protection. As shown at right in Fig 5A, GFP-L254 transcripts were NMD-sensitive when constructs were agroinfiltrated into *N*. *benthamiana*, whereas GFP-TCV transcripts were NMD-resistant.

To determine if other carmovirus 3’ UTRs also confer protection against NMD, 3’ UTRs from four additional carmoviruses were individually inserted downstream of the GFP ORF. The 3’ UTRs of *Cardamine chlorotic fleck virus* (GFP-CCFV), *Carnation mottle virus* (GFP-CarMV) and *Calibrachoa mottle virus* (GFP-CbMV) were all protective, with transcript levels that did not vary significantly in the presence or absence of U1D (Fig 5A, bottom). Only the *Saguaro cactus virus* 3’ UTR (GFP-SCV) was targeted by NMD, with over 2-fold greater accumulation of RNA transcripts with U1D treatment (Fig 5A, bottom). When carmovirus 3’ UTRs were inserted into GFP-L to determine if NMD-resistance persisted in the presence of a longer 3’ UTR, only TCV and CCFV 3’ UTRs maintained their ability to confer NMD resistance (Fig 5B). These data suggest that the CarMV and CbMV 3’ UTRs are NMD-resistant when the 3’ UTR length is short and less susceptible to UPF1 targeting. None of the carmoviruses tested, including CCFV, had more than a few sequential residues in common with TCV in the region just downstream of their CP stop codons (S2A Fig). This suggested that sequence-specific motifs within the TCV or CCFV USR-regions were not responsible for NMD-resistance.

Predicted secondary structures of the 75 nt downstream of the terminating ribosome footprint in the USR-region of carmovirus 3’ UTRs was calculated using RNAslider [49]. TCV and CCFV had the highest minimum folding energies (MFE), indicating their respective regions downstream of the terminating ribosome were the least structured (S3A Fig). Also, these regions in TCV and CCFV had significantly lower GC% content compared to the remaining carmoviruses tested (S3A Fig). SCV, whose 3’ UTR was not capable of conferring resistance even in the context of a shorter 3’UTR, is known to contain a highly structured element in this region required for cap-independent translation called a PTE that spans the border between the CP ORF and the 3’ UTR (S2B Fig) [50].

### Lack of secondary structure and not a specific sequence in the TCV USR is associated with NMD-resistance

**Fig 6:**
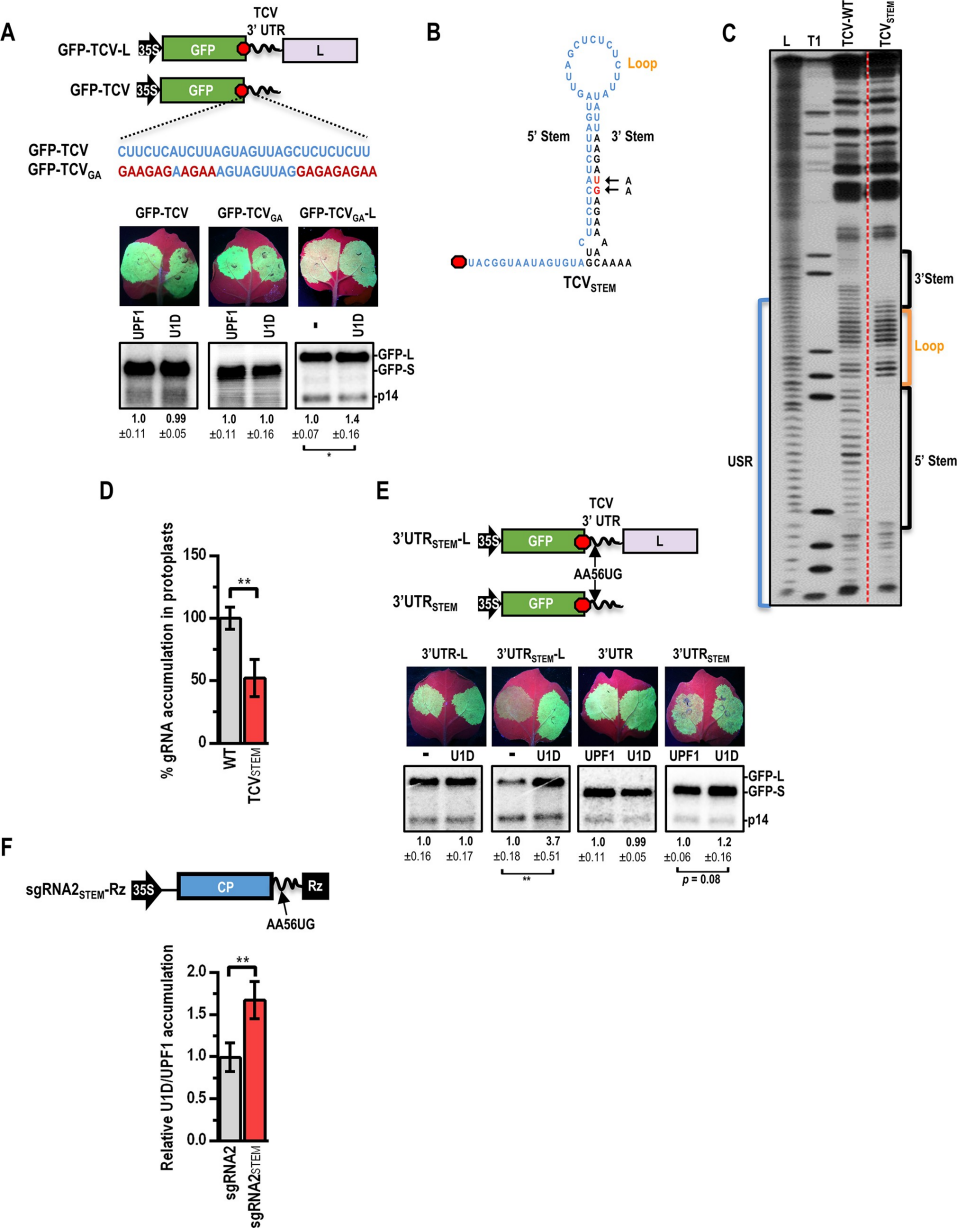
Lack of secondary structure confers NMD-resistance. (**A**) C/U sequences in two USR segments that were mutated to G/A in GFP-TCV_GA_ and GFP-TCV_GA_-L are shown. Reporter constructs were agroinfiltrated into *N*. *benthamiana* along with constructs expressing p14 and either UPF1, U1D, or mock (-). Results are from three infiltrations and standard deviation is shown. See legend to Fig 2B for more information. (**B**) Hairpin generated in USR of TCV_STEM_ is shown. Mutations (in red) were introduced outside of the USR (blue residues) in TCV-WT. (**C**) In-line probing of TCV-WT and TCV_STEM_ USR regions. L, hydroxide-generated ladder; T1, partial RNase T1 digest for position of guanylates. Bands denote residue flexibility (unpaired) required to assume in-line geometry necessary for backbone cleavage. Brackets (right side) denote positions of stem loop formed in TCV_STEM_. (**D**) Accumulation of TCV-WT and TCV_STEM_ transcripts in protoplasts at 40 hpi. gRNA levels were quantified by northern blotting for three independent samples. Error bars denote standard deviation. (**E**) TCV 3’ UTR bearing the 2-base TCV_STEM_ mutation was inserted into either GFP-S or GFP-L reporter (3’UTR_STEM_ or 3’UTR_STEM_-L). 3’UTR-L and 3’UTR_STEM_-L constructs were agroinfiltrated with constructs expressing p14 and either UPF1, U1D, or mock (-). Data are from three infiltrated leaves. Standard deviation is shown. See legend to Fig 2B for more information. (**F**) sgRNA2 and sgRNA2_STEM_ were co-expressed with p14 and either UPF1 or U1D. mRNA accumulation was assayed by RT-qPCR at 72 hpi. RT-qPCR data are from 5 leaves in two independent experiments. mRNA levels were normalized to those of p14 and is presented as the fold change in accumulation with U1D vs. UPF1. Unpaired t test: **p<0.01.

A previous study demonstrated that short PTBP1-binding sites (i.e., pyrimidine tracts) can provide NMD resistance, likely through UPF1 displacement by PTBP1 [23]. Since the TCV USR is pyrimidine-rich (Fig 4A, in blue), it was possible that the USR provided NMD resistance through binding to one of the three *N*. *benthamiana* PTB proteins. To explore this possibility, C/U residues within two possible USR PTB binding sites (CUUCUCAUCUU and CUCUCUCUU) were converted to G/A residues in construct GFP-TCV (3’ UTR of TCV downstream of GFP ORF in GFP-S) or 3’UTR-L (3’ UTR of TCV downstream of GFP ORF in GFP-L) generating GFP-TCV_GA_ and GFP-TCV_GA_-L (Fig 6A, top). Following agroinfiltration, GFP-TCV_GA_ transcripts accumulated to similar levels as GFP-TCV transcripts, indicating that the pyrimidine-rich nature of the USR was not a factor in mediating protection against NMD in the shorter 3’ UTR context (Fig 6A, bottom). The TCV_GA_ insert also conferred significant protection to GFP-L, reducing the previous 3.2-fold increase in the presence of U1D to 1.4-fold (compare GFP-L, Fig 2B with GFP-TCV_GA_-L, Fig 6A). Together, these data suggest the pyrimidine stretches in the TCV USR play only a minor role, if any, in NMD-resistance.

Thirty-one of the USR residues are predicted by the Mfold RNA folding algorithm [51] to form a portion of a weak stem-loop. However, previous in-line RNA structure probing of this region in TCV gRNA fragments showed that nearly every residue of the USR is susceptible to backbone cleavage (i.e., the residues are unpaired and flexible), indicating that the stem-loop is either not forming, or not consistently forming in the RNA fragment population [52] (Fig 6C). In an attempt to stabilize this structure, two point mutations (AA_56_UG) were introduced 5 nt downstream of the USR in full-length TCV-WT and in reporter construct 3’UTR-L, generating TCV_STEM_ and 3’UTR_STEM_-L, respectively (Fig 6B and 6E). These two mutations were predicted to substantially decrease the MFE-75 of this structure (from -9.1 kcal/mol to -17.0 kcal/mol) without altering the sequence of the USR. In-line probing of a 321-nt 3’ terminal fragment from TCV_STEM_ revealed data consistent with generation of this stable stem-loop (Fig 6C). TCV_STEM_ accumulated to only 50% of TCV-WT in protoplasts (Fig 6D), suggesting that formation of this hairpin is detrimental to virus fitness. Importantly, even though the USR sequence was unchanged, NMD-resistance was abolished for transcripts of 3’UTR_STEM_-L (Fig 6E, bottom). When the “L” sequence was removed to give 3’UTR_STEM_, only a 20% increase in RNA accumulation (*p* = 0.08) was observed with U1D expression (Fig 6E, bottom). The modest effect of the STEM mutation could be due to the close-proximity of the poly-A tail to the stop codon in the 3’UTR_STEM_ reporter construct. Earlier studies have demonstrated the NMD-inhibitory effects of PABP binding near PTCs [13–15].

To determine the effect of the STEM mutation in a more natural context (i.e., no poly-A tail), the AA_56_UG STEM mutation was introduced into a sgRNA2 construct with a 3’ ribozyme (Fig 6F). sgRNA2 and sgRNA2_STEM_ were expressed in the presence of UPF1 or U1D, and RNA accumulation was measured by RT-qPCR at 72 hpi. As previously found (Fig 1C), sgRNA2 accumulation did not differ when expressed with UPF1 or U1D. However, sgRNA2_STEM_ accumulation increased 1.7-fold with U1D versus UPF1. This demonstrates that sgRNA2 is inherently NMD-resistant, but becomes sensitive to NMD with the STEM mutations, suggesting that unstructured RNA downstream of the sgRNA2 stop codon is important for NMD-resistance.

### NMD-resistant mRNAs with long 3’ UTRs are significantly more unstructured in the 5’ region of their 3’ UTRs

Previously, the TRAM1, VAMP3, CRIPT, TMED2, and PSMD5 human transcripts were shown to possess NMD-resistant 3’ UTRs that were >1400 nt in length [53]. TRAM1 contained a termination-proximal *cis* element that prevented NMD in mammalian cells due to an unknown mechanism [53]. We hypothesized that unstructured regions of RNA might be proximal to the stop codons in the above transcripts. Interestingly, the MFEs for TRAM1, TMED2, and PSMD5 ranged from -7.5 to -10.4 kcal/mol, which were comparable to the TCV USR (S3B Fig). Furthermore, only VAMP3 contained a GC% over 38.7% in the 75 nt window, suggesting that the majority of these NMD-resistant 3’ UTRs contained A/U-rich, unstructured regions of RNA downstream of their respective stop codons (S3B Fig).

**Fig 7:**
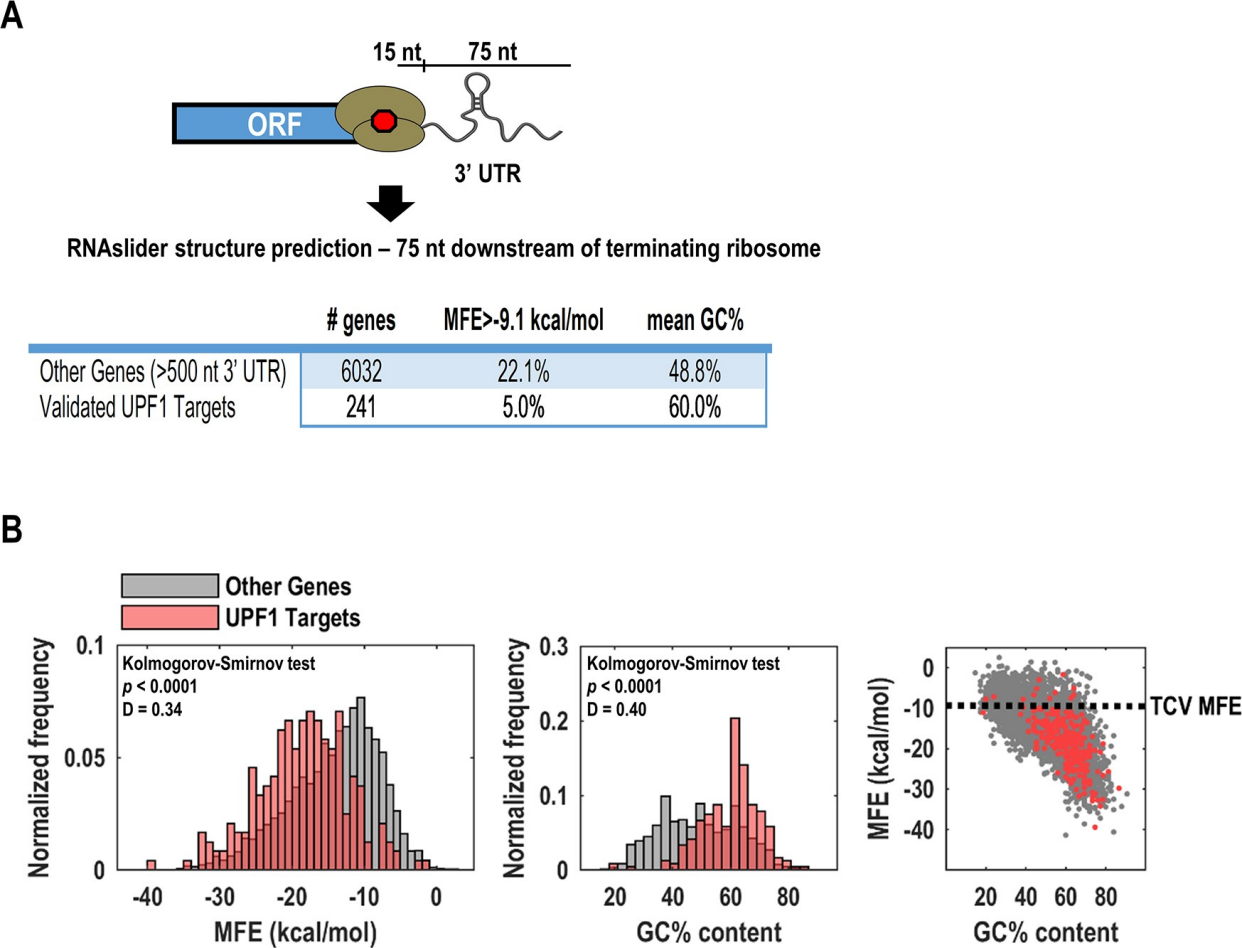
NMD-resistant transcripts contain unstructured regions downstream of stop codons compared to UPF1 targets. (A) Using a previously published dataset that identified NMD-resistant and UPF1-targeted transcripts [36], the minimum folding energies (MFE) for bases 16–90 downstream of each stop codon was calculated using RNAslider [49]. The first 15 bases were omitted to account for the terminating ribosome footprint. The percentage of genes from either NMD-resistant (“other”) or UPF1 targets with MFEs higher than the comparable TCV 3’ UTR region is shown. The mean GC% content for the 75 nt window is listed for both datasets. (B) MFE and GC% content profiles for NMD-resistant and UPF1 targets are shown. The MFE of UPF1 targets was shifted to the left (i.e., more structured) and the GC% content was shifted to the right (i.e., higher GC% content), compared to NMD-resistant transcripts. Dashed line (right panel) indicates the arbitrary cutoff used for the analysis in Fig 7A. The Kolmogorov-Smirnov (KS) test was used to determine if there were significant differences in spread, median, or shape of distributions between NMD-resistant genes and UPF1 targets. The *p* value was calculated from the ‘D’ value, which is the maximum distance between the cumulative distribution functions between the two samples.

To determine if unstructured regions of RNA downstream of termination codons are enriched in human NMD-resistant transcripts, we expanded our analysis to include >6000 NMD-resistant transcripts and 241 validated UPF1 targets that were previously determined by a combination of BRIC-seq, RIP-seq, and CLIP-seq [36]. Using the TCV MFE as an arbitrary cutoff (-9.1 kcal/mol, S3A Fig), 22.1% of NMD-resistant “other genes” contained highly flexible regions downstream of the stop codon (Fig 7A, S1 Dataset). However, only 5.0% of validated UPF1 targets contained a comparable flexible region (Fig 7A, S1 Dataset). The high GC% content of the UPF1 targets is likely responsible for stable secondary structure (Fig 7B). Altogether, these data strongly suggest that unstructured regions of RNA downstream of stop codons can counter NMD.

## Discussion

Multiple studies have established that diverse families of viruses are targeted by NMD [37, 54]. However, given that most published NMD-evasion mechanisms are virus-specific [27, 31–33], it remains unclear if general mechanisms exist, aside from PTBP1 binding [23], that are shared with cellular mRNAs whose otherwise extensive 3’ UTRs would comprise natural targets. For the first time, we have identified features of a virus genome that provide NMD-resistance without sequence specificity, which could be employed across diverse families of viruses and by host mRNAs.

Our results suggest that TCV possesses at least two NMD-resistant genomic features and displays enhanced NMD-resistance compared to the umbravirus PEMV-2 (Fig 1). When the TCV gRNA is translated, ~95% of ribosomes terminate at the p28 stop codon, with the remainder reading through the stop codon to produce the full-length p88 RdRp [41]. Both the TCV RSE readthrough element and the similarly positioned PEMV-2 RSE frameshifting element prevented degradation of an NMD-sensitive GFP reporter (Fig 3). Recoding has previously been shown to stabilize NMD-sensitive transcripts due to displacement of UPF1 by translocating ribosomes [30, 44]. Viruses from diverse families including members of the *Astroviridae*, *Coronaviridae*, *Luteoviridae*, *Tombusviridae*, *Retroviridae*, *Togaviridae*, and *Flaviviridae* utilize ribosome readthrough or frameshifting to produce their replicase proteins [55]. This widespread translation strategy could serve a dual-purpose by simultaneously producing a required protein and protecting an otherwise NMD-sensitive transcript.

The 3’ UTR of TCV only provided NMD-resistance when positioned immediately downstream of an actively translating ORF stop codon (Fig 4B), suggesting that the 3’ UTR would only protect sgRNA2, the template for translation of the CP. The CP is the RNA silencing suppressor [35], which is critical for combatting host antiviral responses, and 180 CP monomers interact to form a single virion, which is required for beetle-vectored transmission between plants [56, 57]. The USR immediately following the CP stop codon was required for NMD-resistance of a GFP construct, and NMD-resistance increased 3-fold when the USR was inserted into a heterologous NMD-sensitive 3’ UTR (Fig 4C). No sequence-specific elements required for NMD-resistance could be identified in the TCV USR, including putative PTB binding sites that have only minor effects on NMD-resistance when mutated (Fig 6A). However, since RNA-binding proteins prefer single-stranded RNA for binding [58], we cannot rule out the possibility that the TCV USR binds to an unidentified protein that antagonizes NMD.

Despite TCV and CCFV 3’ UTRs providing strong NMD-resistance, no sequence similarity was found between their respective USR regions (Figs 5 and S2A). The most noticeable difference between NMD-resistant and NMD-sensitive carmovirus 3’ UTRs was a conserved lack of RNA structure in the NMD-resistant sequences. When a stable hairpin was generated in the USR by introducing a downstream 2-base mutation, NMD-resistance was abolished and virus accumulation was reduced by 50% (Fig 6B–6F). These findings support a link between limited RNA secondary structure, virus fitness, and resistance to NMD. Emerging research has demonstrated that 40S and 80S ribosomes leave footprints in the 3’ UTRs of some host transcripts and 80S are capable of initiating translation [59–61]. It remains to be determined if ribosome footprints in the 3’ UTR correlate with increased NMD resistance. However, we speculate that the association of either ribosomes and/or initiation factors with the 3’ UTR can suppress NMD by occluding UPF1 from the region immediately downstream of the stop codon. Interestingly, plant virus 3’ UTRs (including TCV and CCFV) are highly adapted to recruiting ribosomes and initiation factors to aid cap-independent translation at the 5’ end of the genome [62, 63]. Furthermore, a 116-nt unstructured region upstream of the CP ORF serves as a TCV internal ribosome entry site for early CP translation from the gRNA [39].

Earlier studies that used multiple techniques to identify UPF1-bound transcripts showed that UPF1 binds to sites within 3’ UTRs that are GC-rich, which are predicted to have a higher propensity to form stable secondary structures [8, 20, 36]. Since UPF1 is an RNA helicase, it was proposed that stable secondary structures harbor elevated levels of UPF1 as a result of reduced RNA unwinding activity [20]. NMD-sensitive transcripts also have statistically significant higher 3’ UTR GC% content compared with NMD-resistant transcripts across the human transcriptome [8]. Using a set of previously identified human UPF1 targets (n = 241) and NMD-resistant transcripts (n = 6,032) [36], we showed that highly unstructured regions of RNA downstream of stop codons were over 4-fold enriched in NMD-resistant transcripts versus UPF1 targets (Fig 7).

Transcriptome-wide studies in *Arabidopsis*, yeast, *Drosophila*, and *C*. *elegans* have demonstrated that cellular transcripts show a marked decrease in computationally predicted secondary structure at the termination codon and in the immediate downstream region [64–66]. It has been suggested that unstructured regions serve as a signal for ribosome disassociation from the RNA transcript [66]. It is likely that TCV and possibly many other viruses have evolved to mimic host transcripts by conserving unstructured regions of RNA near some stop codons that may promote proper termination and simultaneously enhance NMD-resistance.

In summary, we propose that TCV uses ribosome recoding to protect its gRNA from NMD, and sgRNA2 has enhanced stability due to the USR. The phenomenon of NMD protection from unstructured RNA immediately downstream of the stop codon may be widespread across viruses and hosts and differs from those of previously described viral NMD-resistance strategies that require sequence-specific RNA stability elements [27] or specific viral proteins [31, 32].

## Methods

### Construction of binary expression plasmids

Binary expression constructs were derived from pBIN61S-GFP and pBIN61S-GFPabc (named GFP-L) [12]. DNA fragments corresponding to 250 nt downstream of the five TCV ORFs were ligated into a unique *BamHI* restriction site immediately following the GFP stop codon of GFP-L. The ΔUSR-L and ΔH5/Pr-L constructs were generated using *BamHI* primers for the corresponding region of the TCV 3’ UTR and ligating into the unique *BamHI* restriction site of GFP-L. The L-3’UTR construct containing the TCV 3’ UTR downstream of the “L” sequence was generated by ligating the 3’ UTR fragment into the unique *XbaI* restriction site immediately following the “L” sequence. Constructs containing carmovirus/umbravirus 3’ UTRs immediately following the GFP ORF were constructed by ligating the respective 3’ UTR fragments into the *BamHI* restriction site following the GFP ORF in pBIN61S-GFP or pBIN61S-GFPabc. GFP-TCV_GA_ was constructed by ligating a synthetic dsDNA fragment (Integrated DNA Technologies) bearing CU-to-GA mutations into the *BamHI* restriction site in pBIN61S-GFP or pBIN61S-GFPabc. Synthetic dsDNA fragments were used to construct the readthrough and frameshift GFP-L binary plasmids by ligating the RSE-L-Pr fragment into the unique *BsaII* and *XbaI* restriction sites of pBIN61S-GFP. Stop codons were introduced upstream of the recoding elements by site-directed mutagenesis of sub-cloned plasmids (pUC19) and were subsequently transferred to the GFP binary plasmid by *BsaI* and *XbaI* restriction digestion and ligation. All GFP constructs contain a *Cauliflower mosaic virus* (CaMV) 35S poly(A) signal following the GFP ORF. Use of the poly(A) signal lengthens the 3’ UTRs by approximately 175-nt. All constructs were sequenced and transformed into *Agrobacterium tumefaciens* strain C58C1.

### Dual-luciferase assay

The RSE_TCV_ or STOP-RSE_TCV_ cassettes were amplified from previously constructed plasmids to introduce *SalI* and *BamHI* restriction sites. Fragments were ligated into the corresponding sites in previously described p2Luc+TCV3’UTR [39] and sequenced for accuracy. RNA transcripts were synthesized using T7 polymerase and were used as templates for *in vitro* translation in wheat germ extracts (WGE) as previously described [39]. Luminescence was measured using the dual-luciferase assay system (Promega) following the manufacturer’s protocol and a Modulus microplate multimode reader (Turner Biosystems).

### Transient NMD assay

The NMD assay has been previously described [12]. Briefly, cultures of *Agrobacterium tumerfaciens* containing binary vectors were cultured in the presence of antibiotics and 20 μM acetosyringone until an OD_600_ of 1.5 was reached. Cultures were resuspended in resuspension buffer (10 mM MgCl_2_, 10 mM MES-K [pH5.6], 100 μM acetosyringone) and mixed for a final OD_600_ of 0.2 for p14, UPF1, and U1D. GFP reporter cultures were mixed for a final OD_600_ of 0.4. Cultures were incubated at room temperature for 2 hours prior to infiltration of *Nicotiana benthamiana* (4^th^ to 5^th^ leaf stage) using a 1 mL syringe. All plants were grown at 24°C with 40–65% relative humidity and a long-day photoperiod (18 h light and 6 h dark). At 48 hpi, GFP fluorescence was observed using a portable long-wavelength UV lamp.

### Viruses and infections

Binary plasmids, which contained the TCV or PEMV-2 genome downstream of a 35S promoter and followed by a HDV ribozyme to produce authentic 3’ ends, were constructed using ligation-independent cloning into the binary pCB301 plasmid [67]. The same strategy was used to place TCV sgRNA1 or sgRNA2 into pCB301. Plasmids were transformed into C58C1 agrobacteria by electroporation. Viral infections were established by infiltrating *Nicotiana benthamiana* with viral binary cultures at a final OD_600_ of 0.4 alongside p14 at an OD_600_ of 0.2. To determine the effects of UPF1 overexpression on TCV replication, UPF1 or U1D were co-infiltrated at a final OD_600_ of 0.2. Infiltrated spots were harvested at 3-days post-infiltration for extraction of RNA or protein.

### Northern and western blotting

RNA was isolated from infiltrated spots using Trizol (Invitrogen). Total RNA was subjected to agarose gel electrophoresis and transferred to a charged nylon membrane by capillary action in 10X MOPS [3-(N-morpholino)propanesulfonic acid]. RNAs were crosslinked to the membrane by UV light and were hybridized with [α-^32^P] dATP-labeled DNA probes targeting GFP, TCV, PEMV-2, or p14 transcripts. Densitometry was used for RNA transcript level quantification using GelQuant.NET software.

Total protein lysates were isolated by resuspending ground leaves in 1X phosphate-buffered saline (PBS) + 2% β-mercaptoethanol. Samples were mixed with 2X Laemmli buffer and boiled. Lysates were resolved by 10% SDS-PAGE and transferred to 0.45 μm nitrocellulose using a semi-dry transfer method. Ponceau S was used to stain for ribulose bis-phosphate carboxylase (Rb) as a loading control. Primary antibody targeting GFP (BioVision, Inc.) or CP was used at a 1:2000 dilution. Secondary antibody (goat anti-rabbit IgG, HRP-conjugated) was used at a dilution of 1:5,000. Enhanced chemiluminescence was detected using SuperSignal West Pico substrate (Thermo Scientific).

### RT-qPCR

Total RNA from infiltrated leaves was treated with RQ1 DNAse (Promega) and used as a template for reverse-transcription, quantitative PCR (RT-qPCR). Primers were designed to amplify 80–200 base fragments of TCV or p14 transcripts, respectively. The SYBR green-based Luna one-step RT-qPCR kit was used following the manufacturer’s protocol (New England BioLabs). For all reactions, p14 transcripts were used as a reference to determine relative gene expression.

### In-line probing

In-line structure probing was performed as previously described [52]. Briefly, fragments containing the TCV 3’ UTR (positions 3732–4053) were end-labelled with [γ-32P]ATP and denatured at 75°C before cooling to 25°C. RNAs were incubated at 22°C in 50 mM Tris-HCl [pH 8.5] and 20 mM MgCl_2_ for 14 h. Samples were separated by 8% polyacrylamide gel electrophoresis (8M urea) alongside a hydroxide-generated RNA cleavage ladder and partial RNase T1 digest. Gels were imaged by autoradiography. At least two independent in-line probing assays were performed for each fragment.

### Predicted secondary structure analysis

Human NMD-resistant transcripts and validated UPF1 targets identified in an earlier study [36] were used for secondary structure analysis. The 3’ UTR sequences of both datasets were extracted from GenBank. NMD-resistant genes with 3’ UTRs <500 nt in length were excluded from the analysis. To examine the predicted secondary structure immediately downstream of the terminating ribosome, bases 16–90 from each 3’ UTR sequence were extracted and used to calculate the minimum folding energy (MFE) with a 75 nt window using RNAslider [49]. GC% content was also calculated in the 75 nt window for each gene. Virus 3’ UTRs and NMD-resistant 3’ UTRs identified by Toma *et al*. [53] were analyzed using the same method. Data is available in S1 Dataset.

## Supporting information

S1 Fig *Tombusviridae* 3’ UTR lengths and GC% content. (**A**) Left, Seven genera of *Tombusviridae* have 3’ UTRs that are longer than the average Arabidopsis 3’ UTR. Red histogram bars denote the number of Arabidopsis genes with a 3’ UTR of a given length. The average lengths of 7 *Tombusviridae* genera 3’ UTRs are labelled. Right, *Tombusviridae* 3’ UTRs shown in the left panel are GC-rich compared with Arabidopsis 3’ UTRs. Blue lines denote the mean GC% content.
(TIF)

S2 Fig Sequence and structure in carmovirus 3’ UTRs. (**A**) Alignment of Carmovirus USR regions. The first 50 bases of 3’ UTRs from carmoviruses were aligned using a progressive method (MATLAB, multialign). Percent sequence similarity and GC% content are shown. (**B**) The RNA secondary structure of the SCV PTE element just past the CP termination codon has previously been elucidated [50]. The minimum folding energy (MFE-75) for bases 16–90 following the CP stop codon is shown.
(TIF)

S3 Fig MFE analysis of carmovirus 3’ UTRs and previously characterized human NMD-resistant 3’ UTRs. (**A**) The MFE for bases 16–90 downstream of each stop codon was calculated using RNAslider [49]. The first 15 bases were omitted to account for the terminating ribosome footprint. The MFE-75 value and GC% content are listed for the five carmoviruses used in this study. The TCV MFE-75 value (-9.1 kcal/mol) was used as an artificial cut-off for an expanded study that included >6000 genes. (**B**) Toma *et al*. [53], previously identified and characterized a set of human mRNAs with long 3’ UTRs that were NMD-resistant. The same MFE analysis was performed on these genes. The MFE-75 value and GC% content (of the 75 nt window) are listed for five NMD-resistant transcripts.
(TIF)

S1 Dataset 3' UTR sequences of select viruses and human transcripts were extracted from GenBank. For each 3' UTR, the GC% content and minimum folding energy (MFE) of bases 16–90 was calculated. See Materials and Methods for details.
(XLSX)
