# Supplementary figures and images for: RNA virus evasion of nonsense-mediated decay
Source: PLoS Pathog. 2018 Nov 19;14(11):e1007459. doi: 10.1371/journal.ppat.1007459 (PMC6277124; doi:10.1371/journal.ppat.1007459)

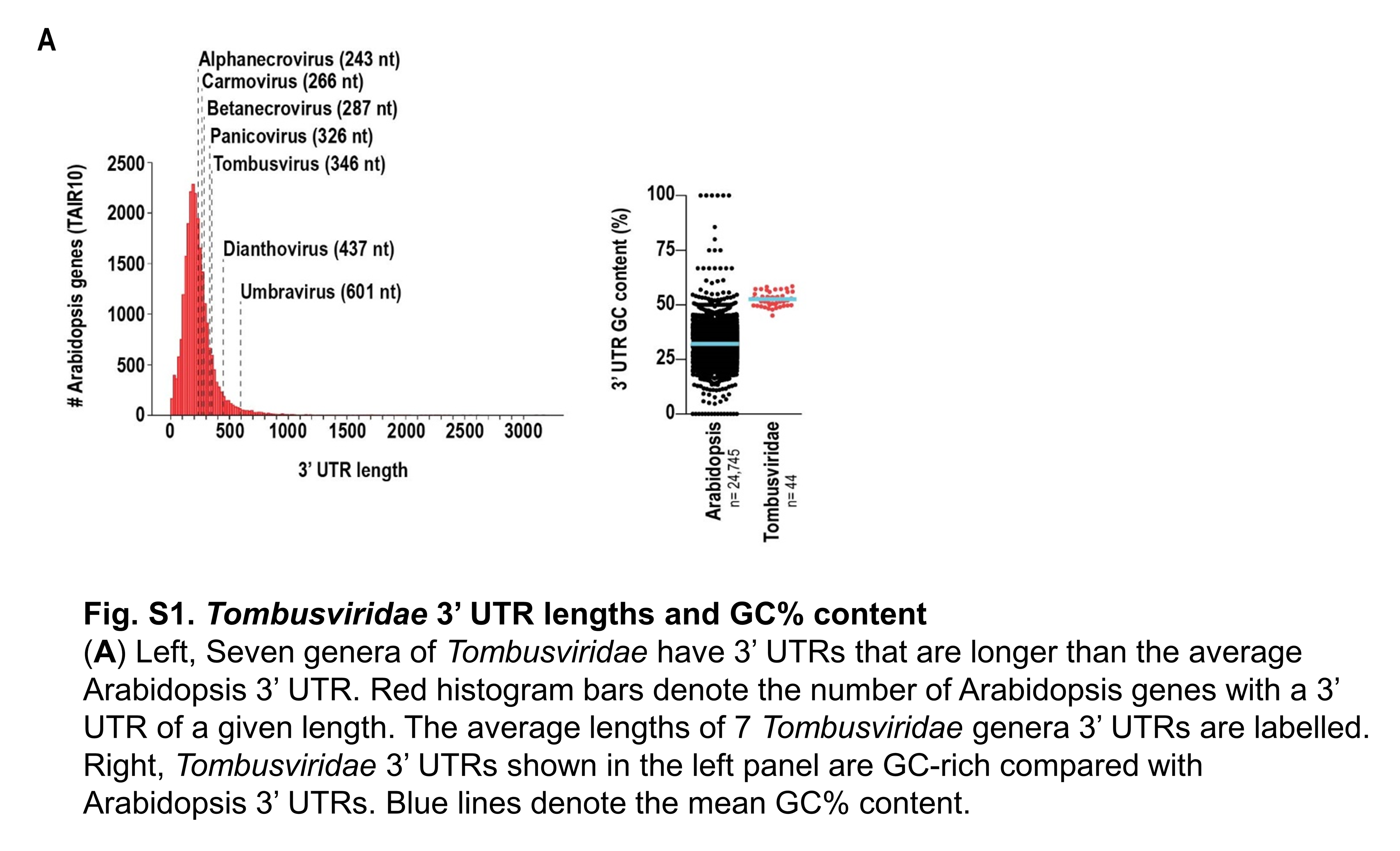

Supplement: S1 Fig — (A) Left, Seven genera of Tombusviridae have 3’ UTRs that are longer than the average Arabidopsis 3’ UTR. Red histogram bars denote the number of Arabidopsis genes with a 3’ UTR of a given length. The average lengths of 7 Tombusviridae genera 3’ UTRs are labelled. Right, Tombusviridae 3’ UTRs shown in the left panel are GC-rich compared with Arabidopsis 3’ UTRs. Blue lines denote the mean GC% content. (TIF) [file ppat.1007459.s001.tif]

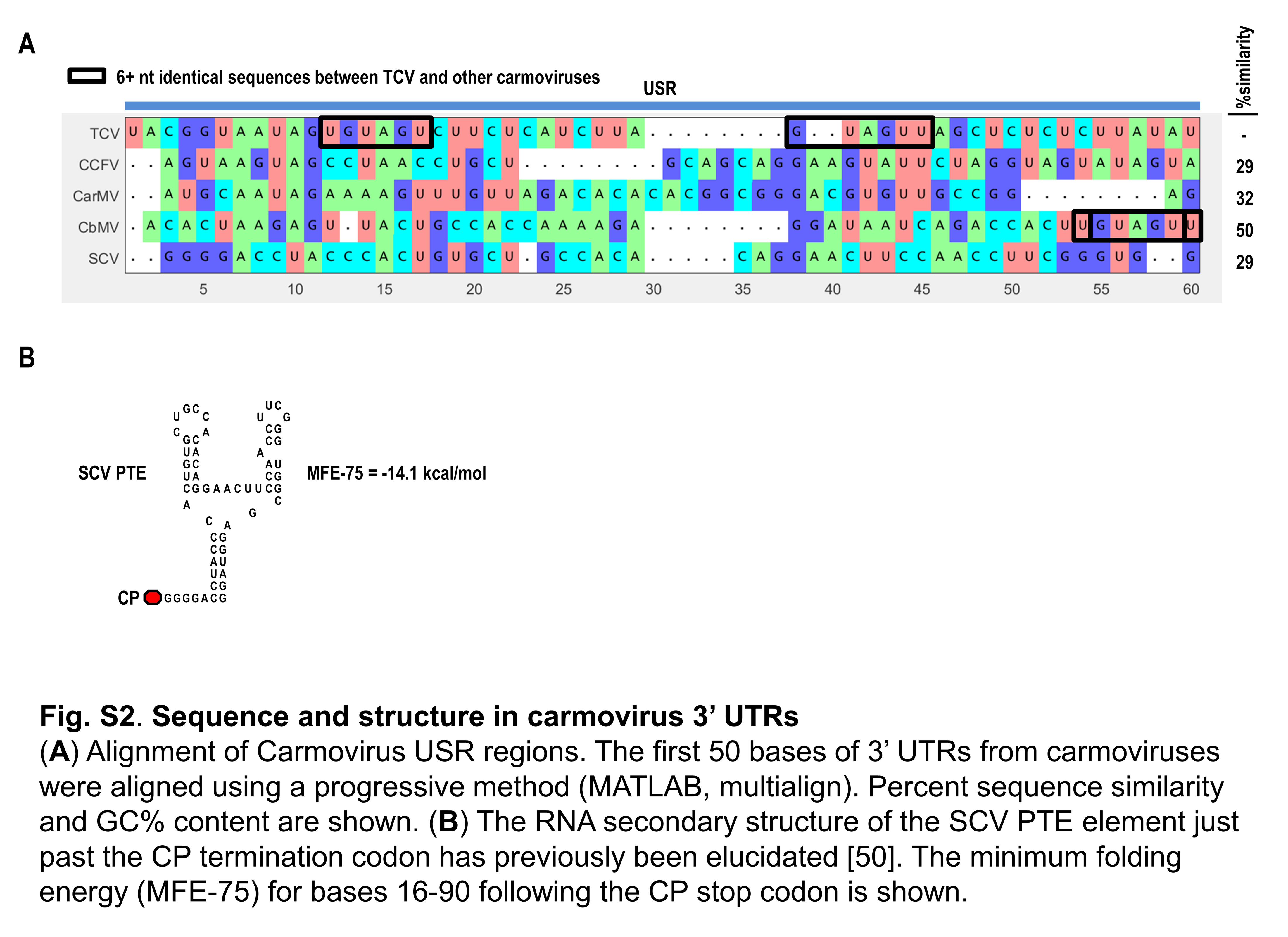

Supplement: S2 Fig — (A) Alignment of Carmovirus USR regions. The first 50 bases of 3’ UTRs from carmoviruses were aligned using a progressive method (MATLAB, multialign). Percent sequence similarity and GC% content are shown. (B) The RNA secondary structure of the SCV PTE element just past the CP termination codon has previously been elucidated [50]. The minimum folding energy (MFE-75) for bases 16–90 following the CP stop codon is shown. (TIF) [file ppat.1007459.s002.tif]

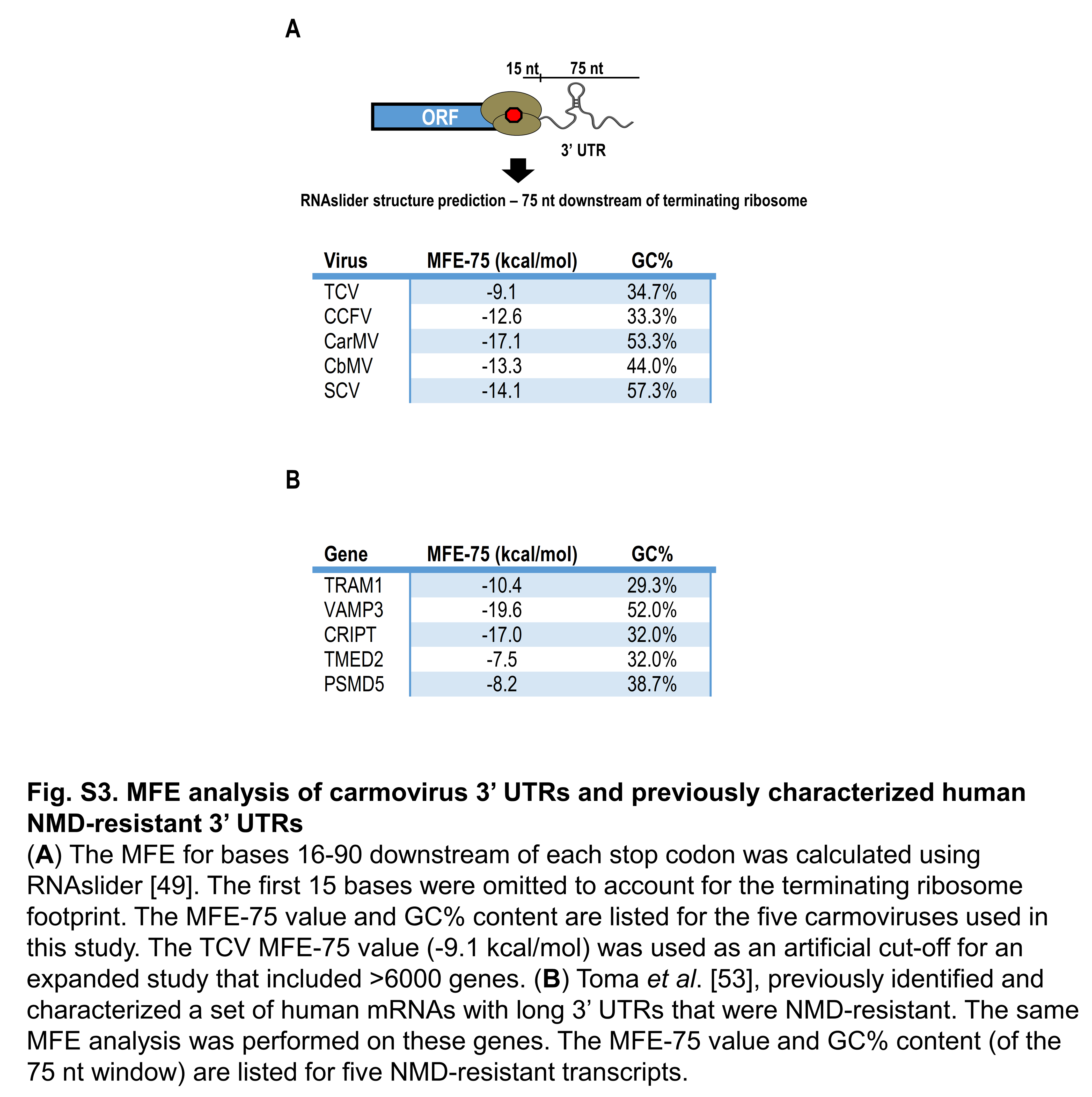

Supplement: S3 Fig — (A) The MFE for bases 16–90 downstream of each stop codon was calculated using RNAslider [49]. The first 15 bases were omitted to account for the terminating ribosome footprint. The MFE-75 value and GC% content are listed for the five carmoviruses used in this study. The TCV MFE-75 value (-9.1 kcal/mol) was used as an artificial cut-off for an expanded study that included >6000 genes. (B) Toma et al. [53], previously identified and characterized a set of human mRNAs with long 3’ UTRs that were NMD-resistant. The same MFE analysis was performed on these genes. The MFE-75 value and GC% content (of the 75 nt window) are listed for five NMD-resistant transcripts. (TIF) [file ppat.1007459.s003.tif]
